# Hybrid Vibration Sensor for Equipment Monitoring and Diagnostics

**DOI:** 10.3390/s24113535

**Published:** 2024-05-30

**Authors:** Ivan V. Bryakin, Igor V. Bochkarev, Vadim R. Khramshin, Vadim R. Gasiyarov

**Affiliations:** 1 Laboratory of Information and Measuring Systems, National Academy of Sciences of the Kyrgyz Republic, Bishkek 720010, Kyrgyzstan; bivas2006@yandex.ru; 2 Department of Electromechanics, Kyrgyz State Technical University Named after I. Razzakov, Bishkek 720010, Kyrgyzstan; elmech@mail.ru; 3 Power Engineering and Automated Systems Institute, Nosov Magnitogorsk State Technical University, 455000 Magnitogorsk, Russia; hvrmgn@gmail.com; 4 Department of Automation and Control, Moscow Polytechnic University, 107023 Moscow, Russia

**Keywords:** vibration-based diagnostics, axial and torsional oscillations, monitored object’s inclination angle, vortex sensor, inclinometer, fluxgate meter, data signal

## Abstract

Vibration diagnostics based on vibroacoustic signal data belong to the most common ways to monitor the technical condition of equipment and technical structures. The paper considers the general issues of vibration-based diagnostics and shows that in general, it is required to monitor both axial and torsional oscillations, as well as the inclination angle, occurring during the operation of various technical objects. To comprehensively monitor these parameters, a hybrid vibration sensor is proposed, simultaneously implementing three operating modes: recording linear displacements of the vibrating object; recording the rotation angle of the object at its torsional oscillations; recording the object angular deviation from the vertical component of the natural local geomagnetic field, i.e., the inclinometer mode. The proposed hybrid sensor design is described, and a theoretical analysis of the sensor’s operation in each of the aforementioned operating modes is performed. The authors show that in the inclinometer mode the sensor actually operates as a fluxgate meter. Generalizing the results of the sensor’s operation simultaneously in all three operating modes, an equation for the total output data signal has been obtained, which allows for obtaining the required information on the current values of linear displacements and rotation and inclination angles by selectively filtering it with respective three filters tuned to specific frequencies. The experimental studies of the proposed hybrid vibration sensor confirmed its ability to record various vibrational disturbances and changes in the inclination angle of the monitored object.

## 1. Introduction

Reliable and efficient equipment operation is determined by many factors and depends on its design, technology, and manufacturing quality, as well as operation conditions, including its structure, load, operating modes, climatic and mechanical impacts, etc. Internal factors such as gradual aging, wear, and corrosion of parts and components also have an impact. Along with these objective factors affecting the equipment performance and reliability, there is a range of subjective factors such as the qualification of maintenance personnel, arrangement of technical maintenance and scheduled works, etc. According to [1], maintenance accounts for 15 to 60% of the total finished product cost while in heavy industry, it may reach 50% of the total production cost. Obviously, all these factors affect equipment operation in totality, and their consideration poses a very complex problem.

Emergencies can be prevented, and equipment reliability can be improved by continuous monitoring of equipment condition, which allows for the timely detection of various faults and defects and arranging appropriate equipment maintenance or repair, thereby preventing grave breakdowns [2,3,4]. Such monitoring allows for taking proper preventive measures, thereby, extending the equipment’s service life [5,6,7].

Vibration-based diagnostics, recognizing the technical condition of machines and mechanisms based on the vibroacoustic signal source data, are among the most common ways of equipment condition monitoring. Vibration inevitably occurs during the operation of any mechanism. Vibration-based monitoring is conduct not only to detect and prevent vibration-related damage but also control the vibroacoustic parameters of equipment to improve its performance [8,9]. Currently, vibration-based analysis is considered one of the best ways for defining the machine condition [10,11]. According to [12], the percentage of vibration-based fault diagnostic techniques exceeds 82%.

Mechanical oscillations are caused by various design and operational factors such as tolerances and clearances, mutual displacements and periodic contacts of individual machine parts, poor balancing and alignment of rotating parts of machines and equipment, and the pulsating load nature [13,14,15]. Even low-amplitude mechanical oscillations frequently induce resonant oscillations in other structural elements, amplifying and becoming a significant source of vibration and noise.

To avoid expenses on eliminating the vibration consequences, reliable vibration measurement devices are required to provide reliable information with the required precision.

## 2. General Issues in Vibration-Based Diagnostics

A vibration analysis for machine monitoring and diagnostics typically comprises three main stages: data collection, signal processing, and fault recognition [16,17,18]. Currently, each of these stages involves a variety of techniques and tools, and choosing the most effective ones may be a rather complex problem since each of them has its own characteristics, advantages, and shortcomings. Obviously, the efficiency of vibration-based diagnostics is first determined by the specifications of primary data collection and processing tools [19,20,21,22].

It is known that any deviation of equipment operating parameters from the norm changes the nature of the interaction of its components and alters the accompanying vibroacoustic processes. In this case, in vibration-based diagnostics, the main physical operating equipment condition data carrier is the vibroacoustic signal, which is a collective concept encompassing information on oscillatory processes (vibrational, hydrodynamic, gas-dynamic, etc.) and acoustic noise of the mechanism in the surrounding environment [21,22,23].

Measuring the vibration of any equipment primarily involves the complex process of obtaining data on the vibration field of its structure. This operating equipment data is read as a set of oscillatory processes, producing the output in the form of certain characteristics of the structure and intensity of these oscillations. The measurement system is capable of recording, functionally converting, and representing the equipment data in a human-readable form according to the measurement task.

**Figure 1:**
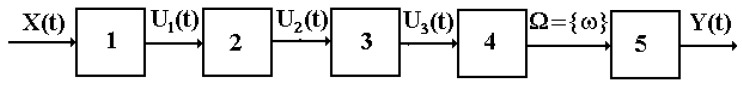
Block Diagram of Vibration Measurement.

In this case, the vibration measurement system is regarded as a unified physical system converting the input signal *X*(*t*) (vibration) into the output one *Y*(*t*) (measured characteristics and parameters) and typically comprising some functional elements connected in series (Figure 1), where 1 is a vibration sensor (transducer) converting mechanical oscillations (vibration) *X*(*t*) into electrical signals *U*_1_(*t*); 2 is an input transducer harmonizing the sensor electrical signal *U*_1_(*t*) with downstream devices and performing various linear transformations (filtration, integration, differentiation); 3 is a linear amplifier, amplifying the input transducer’s electrical output signal *U*_2_(*t*) to the *U*_3_(*t*) magnitude required for further functional conversions and recording; 4 is a functional transducer receiving and calculating the required characteristics and parameters Ω = {ω}; 5 is a recorder displaying the measurement results *Y*(*t*).

In this case, the reliability of the recorded vibration field data primarily depends on the metrological characteristics of vibration sensors detecting and controlling the equipment’s vibration frequency and magnitude in a real-time mode during its operation.

It should be noted that from the variety of parameters characterizing vibration, only those directly or indirectly characterizing the object’s condition are chosen. Based on these parameters, an informatory system of features is formulated for diagnostics. The choice of vibration diagnostic parameters depends on the types of mechanisms under study and the amplitude and frequency range of the measured oscillations [24]. E.g., in the low (0 ÷ 200 Hz), middle (10 Hz ÷ 1 kHz), and high (up to hundreds of kHz) frequency ranges, respectively, the vibration displacement, velocity, and acceleration are most commonly measured.

Vibration displacement is of interest in cases where the object relative displacement or deformation should be known. To find the efficiency of vibrating machines and the impact of vibrations on the human body, vibration velocity is studied while to assess the object’s reliability, vibration acceleration is the primary measured parameter.

It is important to note that two vibration measurement methods are used: 1—kinematic method, where the coordinates of object points relative to a chosen fixed coordinate system are measured, and measuring transducers based on this technique are relative vibration sensors; 2—dynamic method, where in most cases, vibration parameters of an inertial element connected to the object via an elastic suspension are measured relative to an artificial fixed coordinate system, and measuring transducers implementing this technique are absolute vibration sensors or seismic systems.

To sum it up, we can state that the successful solution of vibration measurement problems largely depends on the choice of vibration sensor type, which essentially defines (especially for HF measurements) the vibration signal informativeness and the vibration measurement reliability and accuracy in the operating frequency range. Various types of vibration sensors with different structural designs, based on different physical principles of converting mechanical oscillations into electrical signals, are used in vibration measurement. These primarily include piezoelectric, piezoresistive, inductive, eddy current, and capacitive vibration sensors, measuring and analyzing vibration displacement, velocity, acceleration, etc.

Structurally, by the sensitive element motion type, vibration sensors can be conditionally divided into two classes: 1—axial ones, ensuring linear oscillatory motion of the sensitive element [20,25]; 2—pendulum sensors, implementing angular displacement of the sensitive element during its torsional oscillations relative to the suspension point [26,27].

Furthermore, the occurring vibration may cause dangerous changes in the initial spatial position of process equipment, highly likely leading to catastrophic consequences. Thus, to provide the safe position of special large process equipment (such as pumps, turbines, powerful motors, etc.) relative to their foundations during operation, continuous monitoring of the inclination angle of their functional power elements relative to the Earth’s gravitational field and azimuth (direction) is required. Monitoring the inclination angle of various structures and buildings, e.g., power transmission line poles, is also of high practical importance [28,29].

For this purpose, indirect or direct inclinometers are typically used [30,31]. The first ones use seismic, radar data, successive approximation, and oriented survey data while direct inclinometers use gravity field sensors based on the Earth’s gravity, magnetic fields, telesounding, and gyroscopic effects.

Thus, to effectively address the current challenges in the field of vibration-based diagnostics, the comprehensive use of axial and pendulum vibration sensors is required, as well as inclinometers additionally allowing for direct measurement of changes in the inclination angle of their sensitive elements relative to the vertical component of the Earth’s natural magnetic field.

## 3. The Universal Vibration Sensor (VS) Design Specifics

The aforementioned problem can be effectively addressed by developing a new hybrid vibration sensor with expanded functional capabilities, aimed at combined recording of various types of vibrations and changes in the inclination angle of process equipment and structures, occurring during their operation.

**Figure 2:**
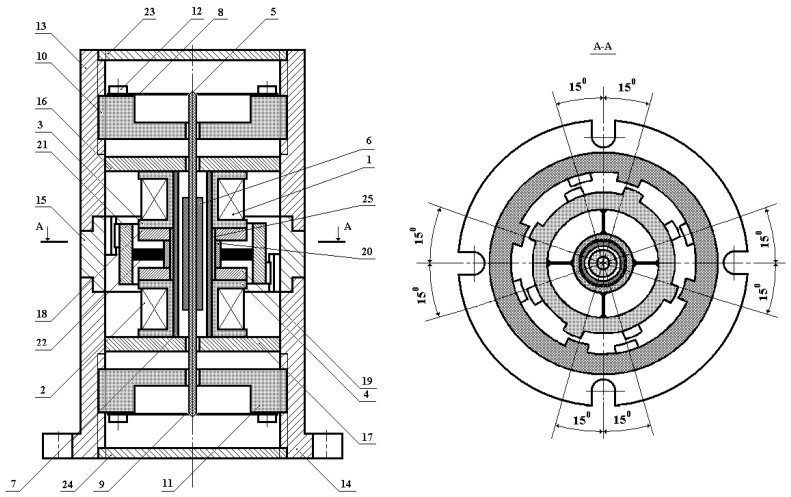
A Hybrid VS Design Version.

Figure 2 shows the structural features of such a vibration sensor version, where 1 and 2 are measuring windings with the number of turns *w*_1_ and *w*_2_, respectively; 3 and 4 are dielectric frames; 5 is a dielectric rod; 6 is a cylindrical ferrite sleeve; 7 is a cylindrical metal electrode; 8 and 9 are elastic suspensions in the form of flat membranes of equal resistance and section, made of beryllium bronze; 10 and 11 are dielectric shaped washers; 12 is a fastening screw; 13 and 14 are, respectively, upper and lower cylinders of the VS housing, made of magnetic material; 15 is an external tooth rim made of magnetic material; 16 and 17 are large washers made of magnetic material; 18 and 19 are small washers made of magnetic material; 20 is a ring made of non-magnetic metal material; 21 is an internal tooth rim made of magnetic material and serving as the inertial element; 22 is elastic elements in the form of flat beams of equal resistance and section, made of beryllium bronze; 23 and 24 are flanges made of non-magnetic metal material; 25 is a thin-walled dielectric sleeve.

Measuring windings 1 and 2 located on, respectively, dielectric frames 3 and 4, form the measuring coils (MC) MC_1_ and MC_2_, based on metal electrode 7. Closing flanges 23 and 24 and upper and lower cylinders 13 and 14, articulated via external tooth rim 15, constitute the VS housing. The cylindrical ferrite sleeve 6, coaxially positioned with respect to electrode 7 on non-magnetic rod 9 mounted on elastic flat suspensions 8 and 9, serves as the sensor’s anchor plunger (AP), functionally acting as its first inertial mass responding to linear accelerations. When the sensor’s inertial mass is spatially linearly displaced relative to the measuring coils MC_1_ and MC_2_, the inductive resistances of these coils change accordingly.

In turn, elastic suspensions 8 and 9 are rigidly fixed with screws 12 on shaped dielectric washers 8 and 9, installed with the possibility of axial travel relative to the sensor housing. At the stage of preparing the sensor for operation, the neutral spatial position of ferrite sleeve 8 relative to measuring coils MC_1_ and MC_2_ is set by displacing washers 10 and 11.

**Figure 3:**
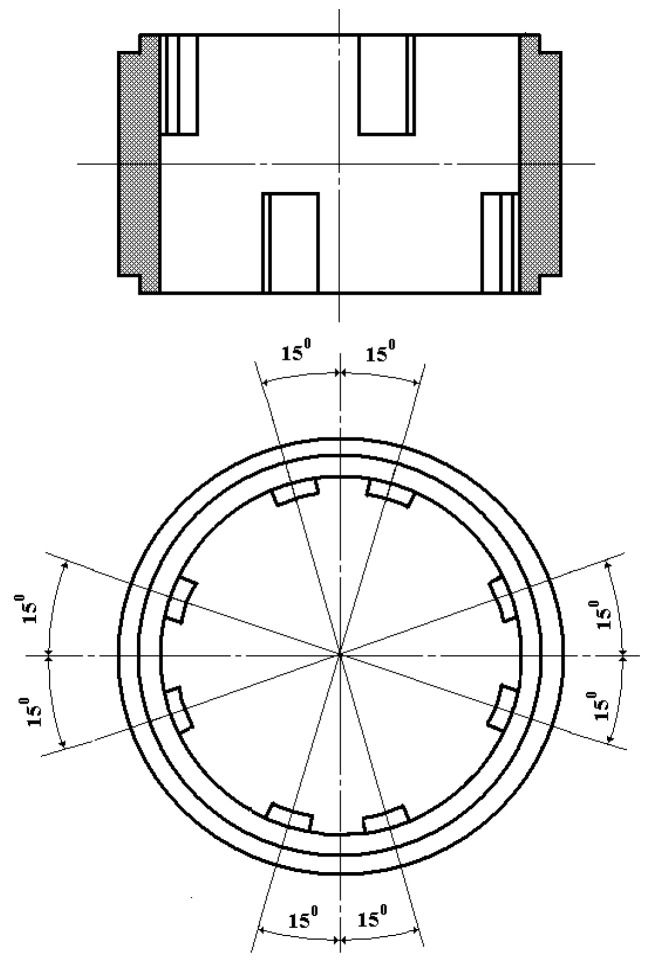
General View of the Internal Tooth Rim.

**Figure 4:**
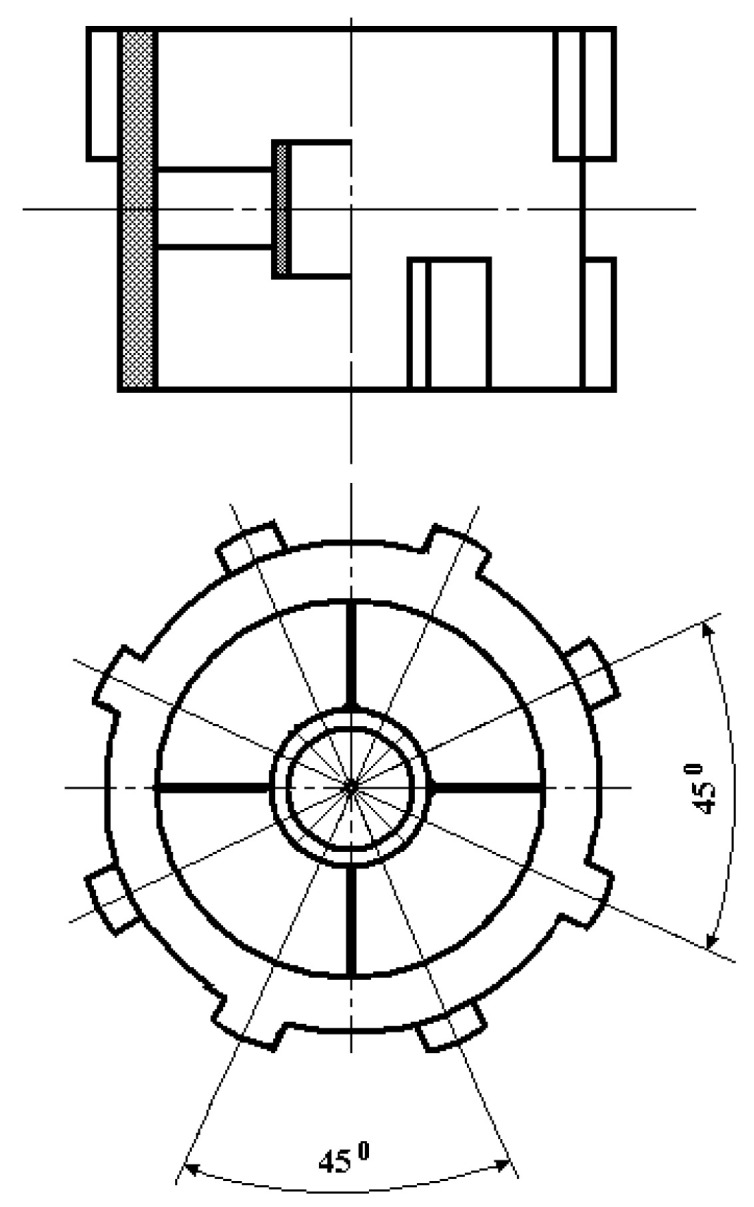
General View of the External Tooth Rim.

Internal and external tooth rims 21 and 15 are, respectively, the rotor and the stator. Internal tooth rim 21 functionally acts as the sensor’s second inertial mass, responding to angular accelerations and connected via four elastic elements 22 to ring 20, fixed through dielectric sleeve 25 on electrode 7 between small washers 18 and 19. Thereat, the lower and upper teeth of internal tooth rim 21 are positioned relative to each other with a spatial angular displacement of 45° (Figure 3) while the lower and upper teeth of external tooth rim 15 are paired relative to each other with a spatial angular displacement of 30° (Figure 4).

The upper and lower teeth of internal tooth rim 21 have such an initial spatial angular displacement relative to the corresponding upper and lower teeth of external tooth rim 15, at which counterclockwise rotation of the rotor relative to the stator causes an increase in the overlap of their upper teeth (reduction of the air gap area) and a corresponding decrease in the overlap of their lower teeth (increase in the air gap area) while clockwise rotation causes a decrease in the overlap of their upper teeth (increase in the air gap area) and an increase in the overlap of their lower teeth (reduction of the air gap area). In this case, at any angular rotor rotation around its axis, the corresponding magnetic resistances of magnetic cores of MC_1_ and MC_2_ will change oppositely.

**Figure 5:**
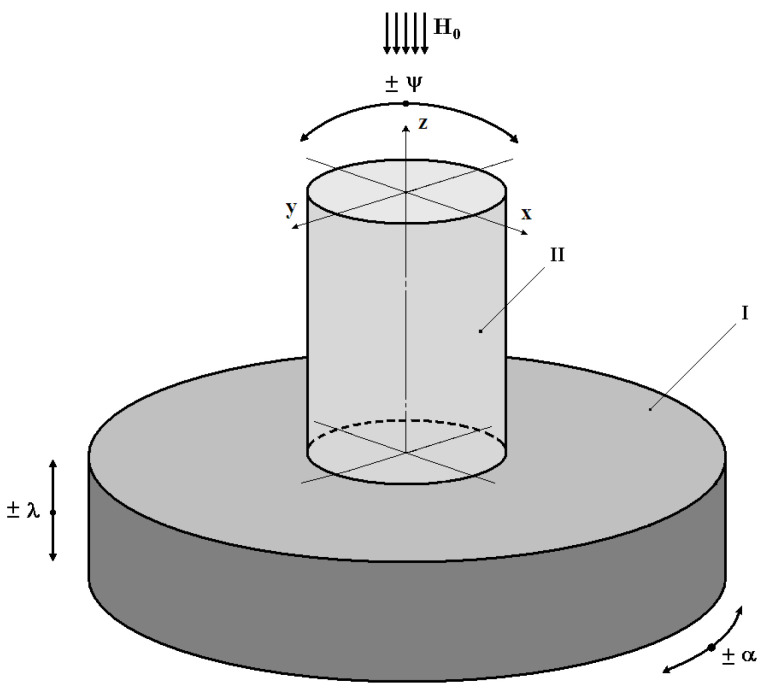
Main Types of Disturbing Impacts.

The proposed VS design version assumes the simultaneous implementation of three VS ‘II’ operating modes: 1—recording the ‘I’ vibrating object’s linear displacements λ relative to the fixed coordinate system; 2—recording the ‘I’ vibrating object’s rotation angle α relative to the fixed coordinate system during its torsional oscillations; 3—recording the angular deviation ψ of the ‘I’ object from the vertical component *H*_0_ of the natural local geomagnetic field, i.e., the inclinometer mode (Figure 5).

It should be noted that the considered VS design version has relatively complex magnetic cores of MC_1_ and MC_2_.

The magnetic core of MC_1_ is formed by the large washer 16, the upper part of ferrite sleeve 6, the small washer 18, the upper parts of inner and outer tooth rims 21 and 15, respectively, and the upper cylinder 13 of the VS housing. Similarly, the magnetic core of MC_2_ is formed by the large washer 17, the lower part of ferrite sleeve 6, the small washer 19, the lower parts of inner and outer tooth rims 21 and 15, respectively, and the lower cylinder 14 of the VS housing.

**Figure 6:**
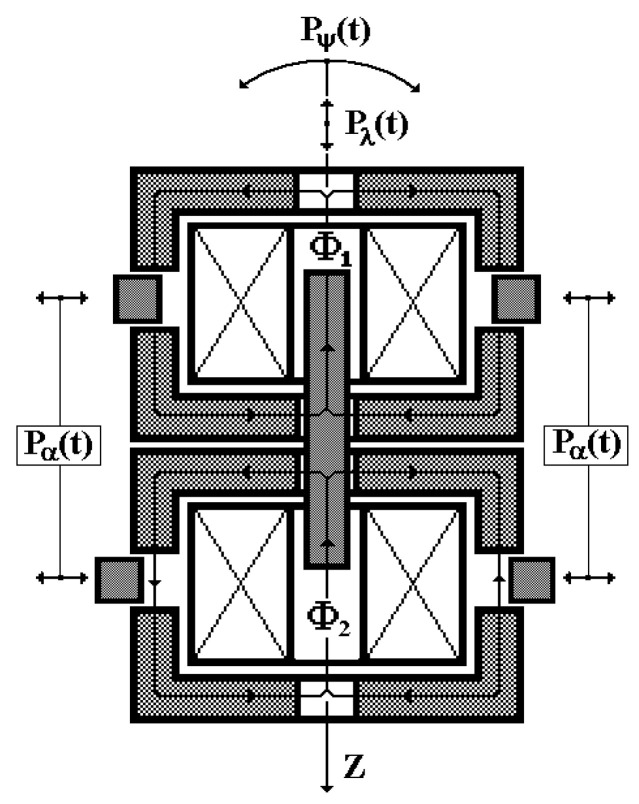
Conditional Layout of VS Magnetic Cores.

In fact, both MC_1_ and MC_2_ have their separate closed magnetic cores, resembling an armored ferrite core in shape, in each of which the excitation currents ${\dot{I}}_{1}$ and ${\dot{I}}_{2}$ of electrical windings MC_1_ and MC_2_ generate, respectively, spatially channeled magnetic fluxes Φ_1_ and Φ_2_ (Figure 6).

Conventionally, magnetic cores of both MC_1_ and MC_2_ can be divided into upper, lower, side, and central sections. Assume that the upper and lower sections of both magnetic cores have virtually the same and time-constant magnetic conductivities.

The central sections of the magnetic cores feature a common movable structural element, capable of simultaneously oppositely varying the magnetic conductivity of both central sections due to the change in the air gap length under the disturbing impact *P*_λ_(*t*) (linear oscillations of the object).

The side sections of the magnetic cores conventionally comprise movable elements capable of independently oppositely varying the magnetic conductivity of these sections due to the change in the air gap area under the disturbing impact *P*_α_(*t*) (rotational oscillations of the object).

Under the disturbing impact *P*_ψ_(*t*) (deviations of the VS housing from the vertical axis *Z* of the absolute coordinate system), the projection of the vertical component of the natural local geomagnetic field onto the VS’ vertical axis of symmetry changes correspondingly.

When sudden disturbing forces affect the object or change, transient vibration occurs, which may change slowly or rapidly, e.g., in the form of increasing or decreasing oscillatory or non-oscillatory motion or pulses of various shapes. Transient vibration has a continuous spectrum and can be described by a Fourier integral.

(1) $$S(\omega )=\int_{-\infty }^{+\infty }[\zeta (t)\times e^{-j\omega t}\times dt].$$

The spectrum occupies the entire frequency range from ω = 0 to ω = ∞, indicating the presence of transient vibration amplitudes at any frequency in the spectrum.

It should be noted that despite the simultaneous implementation of all three VS operating modes, to understand the specifics of the VS operation principles more comprehensively, each of the three specified operating modes will be analyzed separately with the corresponding single disturbing factor applied.

## 4. Analyzing the VS Operation in the Linear Displacement λ Recording Mode

Consider the VS operation specifics in the mode of recording the object’s linear oscillatory displacements λ along the vertical axis Z of the absolute coordinate system.

In this case, the VS can be represented as a differential inductive transducer with a variable air gap length and a pass-through AP.

**Figure 7:**
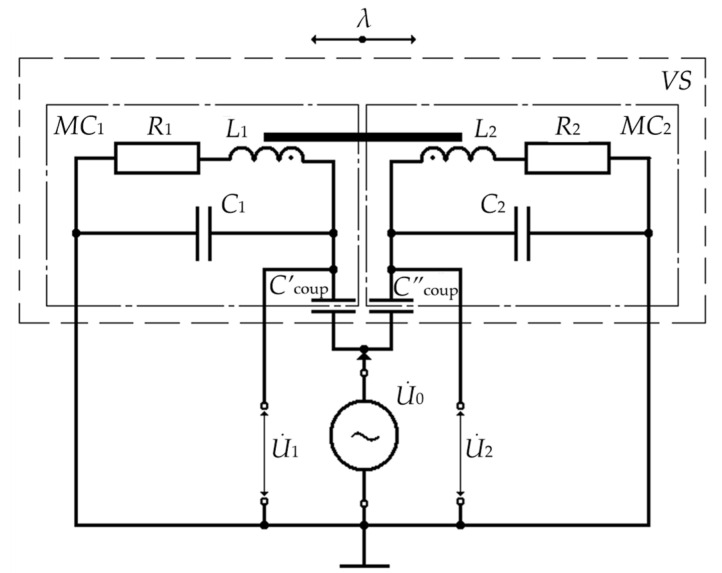
The VS Equivalent Electrical Circuit.

The specifics of the considered VS are that electrical windings of MC_1_ and MC_2_ are designed with maximum possible structural identity, and the lower rows of these electrical windings, together with electrode 7, form respective two-electrode cylindrical coupling capacitors $C_{coup}^{\prime }\;and\;C_{coup}^{\prime \prime },$ with which electrical windings of MC_1_ and MC_2_ form the first and second independent resonant half-bridge *LC* converters relative to the housing (see Figure 7).

In Figure 7, $C_{coup}^{\prime }=C_{coup}^{\prime \prime }$; *w*_1_ = *w*_2_ = *w* and *l*_1k_ = *l*_2k_ = *l*_k_ are, respectively, the number of turns and the length of electrical windings of MC_1_ and MC_2_ (structural parameters); *L*_1_ and *L*_2_, *R*_1_ = *R*_2_ = R*, and *C*_1_ = *C*_2_ = C* are, respectively, inductances, active resistances, and stray capacitances of electrical windings of MC_1_ and MC_2_ (electrical parameters); ${\dot{U}}_{0}$, ${\dot{U}}_{1}$, and ${\dot{U}}_{2}$ are, respectively, the power voltage of the half-bridge *LC* converters from the harmonic signal generator and electrical voltages from the outputs of the first and second *LC* converters.

The following power voltage is applied to the electrical windings of MC_1_ and MC_2_ through the coupling capacitors $C_{coup}^{\prime }\;and\;C_{coup}^{\prime \prime }$ from a special sine-wave voltage generator:

(2) u0(t)=U0×sinω0t=Im[U˙0×ejω0t],

where $U_{0}$ and ω_0_ are, respectively, the power voltage amplitude and angular frequency.

In turn, data signals from the output terminals of lower rows of electrical windings of MC_1_ and MC_2_ are recorded as respective voltages ${\dot{U}}_{1}$ and ${\dot{U}}_{2}$, the difference of which $\Delta \dot{U}={\dot{U}}_{1}-{\dot{U}}_{2}$ is taken as the measured value.

At the linear axial displacement of ferrite sleeve 6 inside MC_1_ and MC_2_, their inductances *L*_1_ and *L*_2_ change proportionally to the ferrite sleeve 6 mass portions located inside the respective measuring coils. In this case, these inductances are determined as follows:

(3) $$L_{1}=\mu \times w^{2}\times (D_{2}-D_{1})\times \frac{\left(l_{0}+\delta \right)}{l_{k}}=L_{0}+\Delta L;\;L_{2}=\mu \times w^{2}\times (D_{2}-D_{1})\times \frac{\left(l_{0}-\delta \right)}{l_{k}}=L_{0}-\Delta L,$$

where *w* and *l_k_* are, respectively, the number of turns and the length of measuring coils; *D*_2_ and *D*_1_ are outer and inner diameters of the measuring coil cross-section in centimeters; *l*_0_ is the ferrite sleeve 6 embedding value in centimeters; δ is the linear axial displacement of ferrite sleeve 6 under the impact of external disturbances *P*_λ_(*t*); μ is the magnetic permeability of the ferrite sleeve 6 material; *L*_0_ is the measuring coil inductance at the neutral position of ferrite sleeve 6; Δ*L* is the change in inductance under the impact of external disturbances *P*_λ_(*t*).

With the neutral AP position, the reactance of MC_1_ and MC_2_ is determined by the following equations:

(4) $$L_{1}=L_{2}=L_{0};\;X_{L_{1}}=X_{L_{2}}=X_{L0},$$

where $X_{L_{1}}=\omega_{0}\times L_{1}$ and $X_{L_{2}}=\omega_{0}\times L_{2}$ are, respectively, the current MC_1_ and MC_2_ reactance values; $X_{L0}$ is the MC_1_ and MC_2_ reactance value at the neutral AP position, i.e., when the bridge circuit is balanced.

Thus, in the balanced state mode when $X_{L_{1}}=X_{L_{2}}=X_{L0}$, for the measured $\Delta \dot{U}$ value, we can write:

(5) $$\Delta \dot{U}={\dot{U}}_{1}-{\dot{U}}_{2}=0,$$

where ${\dot{U}}_{1}={\dot{U}}_{0}\times \frac{R_{1}+j\times X_{L_{1}}}{R_{1}+j\times (X_{L_{1}}-X_{C_{coup}^{\prime }})}+{\dot{U}}_{CMI}$; ${\dot{U}}_{2}={\dot{U}}_{0}\times \frac{R_{2}+j\times X_{L_{2}}}{R_{2}+j\times (X_{L_{2}}-X_{C_{coup}^{\prime \prime }})}+{\dot{U}}_{CMI}$; ${\dot{U}}_{CMI}$ is the complex value of additive common-mode interferences.

When an external impact is applied to the AP, causing its linear axial displacement δ, reactances change correspondingly by $\pm \Delta X_{L}$.

Then, given the aforementioned, for the reactance of MC_1_ and MC_2_, we can write:

(6) $$X_{L_{1}}=\omega \times L_{1}=\omega \times L_{0}+\omega \times \Delta L=X_{L0}+\Delta X_{L};\;X_{L_{2}}=\omega \times L_{2}=\omega \times L_{0}+\omega \times \Delta L=X_{L0}-\Delta X_{L}.$$

According to Equations (5) and (6), the recorded voltages ${\dot{U}}_{1}$ and ${\dot{U}}_{2}$:

(7) $${\dot{U}}_{1}={\dot{U}}_{0}\times \frac{R^{*}+j\times (X_{L_{0}}+\Delta X_{L})}{R^{*}+j\times (X_{L_{0}}+\Delta X_{L}-X_{C^{*}})}+{\dot{U}}_{CMI\;}\;and\;{\dot{U}}_{2}={\dot{U}}_{0}\times \frac{R^{*}+j\times (X_{L_{0}}-\Delta X_{L})}{R^{*}+j\times (X_{L_{0}}-\Delta X_{L}-X_{C^{*}})}+{\dot{U}}_{CMI},$$

where R* = *R*_1_ + *R*_2_.

Considering that the inductive-capacitive half-bridges are powered by voltage at the cyclic series-resonance frequency $\omega_{0}=\omega_{p}=1/\sqrt{L_{0}\times C^{*}}$ of their electrical circuits $L_{1};\;C_{coup}^{\prime }\;and\;L_{2};\;C_{coup}^{\prime \prime }$, and the reactance of coupling capacitors $C_{coup}^{\prime }\;and\;C_{coup}^{\prime \prime }$ is determined by the equation $X_{C_{cв}^{\prime }}=X_{C_{cв}^{\prime \prime }}=X_{C^{*}}={\left(\omega_{0}\times C^{*}\right)}^{-1}$, we can accept the following:

$$X_{L0}=X_{C^{*}}$$

Then, Equation (7) are easily transformed as follows:

(8) $${\dot{U}}_{1}={\dot{U}}_{0}\times \frac{R^{*}+j\times (X_{L_{0}}+\Delta X_{L})}{R^{*}+j\times \Delta X_{L}}+{\dot{U}}_{CMI}\;and\;{\dot{U}}_{2}={\dot{U}}_{0}\times \frac{R^{*}+j\times (X_{L_{0}}-\Delta X_{L})}{R^{*}-j\times \Delta X_{L}}+{\dot{U}}_{CMI}.$$

For the recorded measured $\Delta \dot{U}$ value, after simple mathematical transformations, we obtain the following equations:

(9) $$\dot{U}={\dot{U}}_{1}-{\dot{U}}_{2}={\dot{U}}_{0}\times \frac{R^{*}+j\times \left(X_{L_{0}}+\Delta X_{L}\right)}{R^{*}+j\times \Delta X_{L}}+{\dot{U}}_{CMI}-{\dot{U}}_{0}\times \frac{R^{*}+j\times (X_{L_{0}}-\Delta X_{L})}{R^{*}-j\times \Delta X_{L}}+{\dot{U}}_{CMI}=\;={\dot{U}}_{0}\times \frac{2\times (X_{L_{0}}+R^{*})\times \Delta X_{L}}{(R^{*}{)}^{2}+(\Delta X_{L}{)}^{2}}-{\dot{U}}_{0}\times j\times \frac{2\times R^{*}\times \Delta X_{L}}{(R^{*}{)}^{2}+(\Delta X_{L}{)}^{2}}.$$

Considering that ${\left(R^{*}\right)}^{2}$ >> ${\left(\Delta X_{L}\right)}^{2}$, Equation (9) can be reduced to the form:

(10) ΔU˙=U˙0×2×(XL0+R*)(R*)2×ΔXL−U˙0×j×2R*×ΔXL=Re(ΔU˙)−j⋅Im(ΔU˙),

where Re(ΔU˙)=ΔU˙×cos(argΔU˙) and Im(ΔU˙)=ΔU˙×sin(argΔU˙) are, respectively, the real and imaginary components of the VS’ difference output voltage $\Delta \dot{U}$.

The analysis of the obtained Equation (10) shows that the imaginary component gives more specific information on the AP’s response to an external disturbance:

(11) Im(ΔU˙)=−U˙0×2R*×ΔXL.

To improve the accuracy of measuring the imaginary component of the VS’ output difference voltage, it is proposed to use synchronous detection, where the synchronous detector (SD) is a linear three-port device with the conductivity, alternating synchronously with the power voltage frequency due to a commutation vector. In general, we can write the equation for a relay full-wave SD as follows:

(12) $$u_{OUT}(t)=\Delta u(t)\times u_{C}(t),$$

where $u_{OUT}\left(t\right)$ is the SD’s output value; Δu(t) is the measured value; $u_{C}(t)$ is the commutation vector determining the SD’s conductivity change pattern.

To obtain a scalar component of a vector $\Delta \dot{U}$ value, proportional to its projection onto the imaginary coordinate axis, we will use the simplest relay full-wave quadrature SD with a commutation vector $u_{C}(t)=U_{C}\times sign(sin\omega_{0}t)$ that is in phase with the generator power voltage ${\dot{U}}_{0}$.

When superimposing the SD’s commutation vector and the power voltage ${\dot{U}}_{0}$ vector onto the imaginary coordinate axis on the complex plane, the constant component of the output voltage ${\dot{U}}_{0}$ of the SD operating in the switching mode will be determined as follows:

(13) $$\Delta U_{ID}=K_{ID}\times \left|\Delta \dot{U}\right|\times \sin(\arg\Delta \dot{U})=-K_{ID}\times \frac{1}{R^{*}}\times \Delta X_{L}\times \cdot U_{0},$$

where Δ*U_p_* is the recorded effective value of the reactive component of the SD’s output voltage $\Delta \dot{U}$; *K*_I*D*_ is the SD’s conversion factor; $\left(\arg\;\Delta \dot{U}\right)$ is the phase angle between the SD’s input voltage $\Delta \dot{U}$ and the commutation vector.

Equation (13) shows that the use of relay-type SD will allow not only determining the corresponding measured signal vector component but also completely eliminate the impact of higher harmonics and interference superimposed on the measured voltage.

In general, the basic dynamics equation for a VS with relative linear displacement δ(*t*) of the sensitive ferrite sleeve 6 element and the VS housing can be represented as follows:

(14) $$\frac{d^{2}\delta }{{dt}^{2}}+2\times \Omega_{1}\times \beta_{0}\times \frac{d\delta }{dt}+\Omega_{1}^{2}\times \delta =-\frac{d^{2}\lambda }{dt^{2}},$$

where λ is the linear axial displacement of the VS housing in the absolute coordinate system; $\Omega_{1}=\sqrt{{W_{1}/M}_{1}}$ is the VS cyclic eigenfrequency; *W*_1_ is the stiffness of elastic elements 8 and 9 (Figure 2), *M*_1_ is the mass of inertial element 6 (Figure 2); β_1_ is the attenuation degree.

Then, according to (2) and (14), we have:

(15) $$\Delta L(t)=\mu \times w^{2}\times (D_{2}-D_{1})\times {\left(l_{k}\right)}^{-1}\times \delta (t).$$

Considering Equations (11) and (15) and given the aforementioned, for the imaginary component of the measuring bridge output voltage, we can write:

(16) $$\Delta U_{ID}(t)=-\omega_{0}\times K_{ID}\times \mu \times w^{2}\times (D_{2}-D_{1})\times {\left(l_{k}\right)}^{-1}\times {\left(R^{*}\right)}^{-1}\times \cdot U_{0}\times \delta (t)=-\omega_{0}\times K_{ID}\times Q\times \cdot U_{0}\times U_{K}\times \delta (t),$$

where $Q=\mu \times w^{2}\times (D_{2}-D_{1})\times {\left(R^{*}\times l_{k}\right)}^{-1}$ is the VS design factor;

From Equation (16), the required δ(*t*) parameter is:

(17) $$\delta (t)=-\Delta U_{ID}(t)\times {\left(\omega_{0}\times K_{ID}\times Q\times \cdot U_{0}\right)}^{-1}.$$

In fact, Equation (17) determines the δ(*t*) change pattern, according to which, using the solution to the differential Equation (3), the acceleration $\frac{d^{2}\lambda }{dt^{2}}$ of the VS housing displacement in the absolute coordinate system is ultimately found.

In general, according to (1), the disturbing impact *P*_λ_(*t*) on an object causes a complex oscillatory nature of its linear displacement:

(18) $$\lambda (t)=\sum_{i=1}^{n}\lambda_{i}\times \sin(\Omega_{i}t+\varphi_{i}).$$

Since the ferrite sleeve inertial mass together with the elastic suspension forms a high-Q resonant mechanical system, at the disturbing impact *P*_λ_(*t*), the inertial mass oscillation will be excited at the resonant frequency $\Omega_{I}$ of this mechanical system.

Therefore, at a sudden disturbing impact *P*_λ_(*t*) on an object, free inertial mass oscillations arise, which can be conventionally represented as follows:

(19) $$\delta (t)=\delta_{\max}\times \sin(\Omega_{I}t+\varphi_{\delta }).$$

where *δ*_max_ is the maximum AP displacement relative to its neutral position; Ω_I_ is the resonant angular core displacement frequency; φ_δ_ is the initial AP displacement phase.

In this case, for the recorded data signal, we can write:

(20) ΔUID(t)=−ω0×KID×Q×⋅U0×Imδ˙max×ejΩIt.

The resulting Equation (20) reflects a clearly expressed functional dependence of the increment of the measuring bridge output voltage imaginary component on the AP linear displacement caused by external vibrations, the parameters of the temporary implementation of which indirectly reflect the processes occurring in the environment.

## 5. Analyzing the VS Operation in the Rotation Angle α Recording Mode

Consider the specifics of the VS operation in the mode of recording the rotation angle α of the vibrating object ‘I’ relative to the fixed coordinate system at its torsional oscillations.

**Figure 8:**
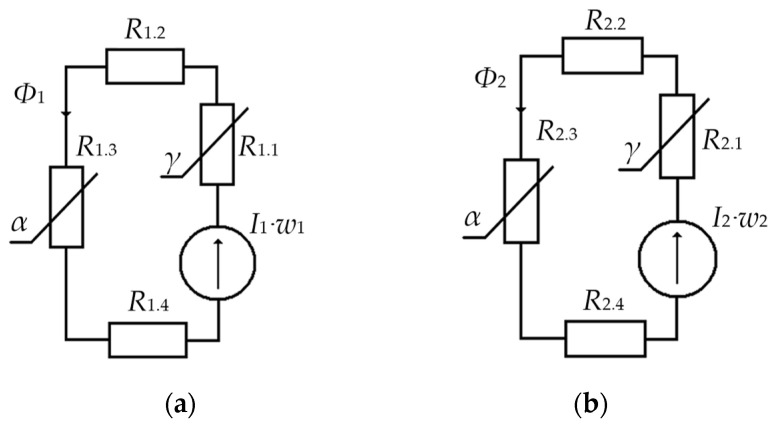
Equivalent Circuits of the VS Upper (**a**) and Lower (**b**) Magnetic Cores.

To do this, we will consider versions of equivalent circuits of the magnetic cores of MC_1_ and MC_2_, formed by a closed magnetic circuit of corresponding sections with lumped parameters (Figure 8), where *R*_1.1_ and *R*_2.1_ are, respectively, the total magnetic resistances of the central sections of the magnetic cores of MC_1_ and MC_2_; *R*_1.2_ and *R*_2.2_ are, respectively, the total magnetic resistances of the upper sections of the magnetic cores of MC_1_ and MC_2_; *R*_1.3_ and *R*_2.3_ are, respectively, the total magnetic resistances of the side sections of the magnetic cores of MC_1_ and MC_2_; *R*_1.4_ and *R*_2.4_ are, respectively, the total magnetic resistances of the lower sections of the magnetic cores of MC_1_ and MC_2_; ${\dot{F}}_{1}={\dot{I}}_{1}\times w_{1}$ and ${\dot{F}}_{2}={\dot{I}}_{2}\times w_{2}$ are, respectively, magnetomotive forces in the closed magnetic cores of MC_1_ and MC_2_, created by currents in MC_1_ and MC_2_; ${\dot{\Phi }}_{1}$ and ${\dot{\Phi }}_{2}$ are, respectively, the magnetic fluxes in the magnetic cores of MC_1_ and MC_2_.

The differential equation describing the VS operation in the rotation angle α recording mode can be represented as follows:

(21) $$\frac{d^{2}\theta }{{\mathrm{dt}}^{2}}+2\times \Omega_{2}\times \beta_{1}\times \frac{d\theta }{\mathrm{dt}}+\Omega_{2}^{2}\times \theta =-\frac{d^{2}\alpha }{{\mathrm{dt}}^{2}}.$$

where α is the VS housing rotation angle around its vertical axis of symmetry in the absolute coordinate system; θ is the rotation angle of the annular sensing element in the form of an internal tooth rim 21 relative to the VS housing (Figure 1); $\Omega_{2}=\sqrt{{W_{2}/J}_{2}}$ is the VS cyclic eigenfrequency; *W*_2_ is the angular stiffness factor of elastic elements 22 (Figure 2), *J*_2_ is the inertia moment of the annular sensitive element in the form of an internal tooth rim 21 (Figure 2); β_2_ is the damping ratio.

For this VS operating mode, we assume that the VS is only affected by a single disturbing factor *P*_α_(*t*) causing torsional VS oscillations around its vertical axis of symmetry. In this case, the values of lumped parameters *R*_1.1_, *R*_1.2_, *R*_1.4_ and *R*_2.1_, *R*_2.2_, *R*_2.4_ of, respectively, sections of the magnetic cores of MC_1_ and MC_2_ can be taken constant. In other words, in this case, the informative parameters are the total magnetic resistances *R*_1.3_ and *R*_2.3_ of respectively, the central sections of the magnetic cores of MC_1_ and MC_2_, the values of which are functionally directly related to the disturbing factor *P*_α_(*t*).

Under the impact of electric currents ${\dot{I}}_{1}$ and ${\dot{I}}_{2}$ of, respectively, MC_1_ and MC_2_, corresponding magnetomotive forces are created in their magnetic cores:

(22) $${\dot{F}}_{1}=w_{1}\times {\dot{I}}_{1}=\sum_{m-1}^{4}{\dot{F}}_{1.m}\;\mathrm{and}\;{\dot{F}}_{2}=w_{2}\times {\dot{I}}_{2}=\sum_{m-1}^{4}{\dot{F}}_{2.m}.$$

where ${\dot{F}}_{1.m}$ and ${\dot{F}}_{2.m}$ are local magnetomotive forces in the corresponding sections of the magnetic cores of MC_1_ and MC_2_.

In turn, magnetomotive forces initiate the generation of magnetic fluxes ${\dot{\Phi }}_{1}$ and ${\dot{\Phi }}_{2}$ in the respective magnetic cores of MC_1_ and MC_2_:

(23) $${\dot{\Phi }}_{1}=\frac{{\dot{F}}_{1}}{R_{M1}}=\frac{{\dot{F}}_{1}}{\sum_{m-1}^{4}R_{1.m}}\;\mathrm{and}\;{\dot{\Phi }}_{2}=\frac{{\dot{F}}_{2}}{R_{M2}}=\frac{{\dot{F}}_{2}}{\sum_{m-1}^{4}R_{2.m}},$$

where $R_{M2}=\sum_{m-1}^{4}R_{2.m}$ and $R_{M1}=\sum_{m-1}^{4}R_{1.m}$ are the total magnetic resistances of, respectively, the magnetic cores of MC_1_ and MC_2_.

Given the aforementioned, for the considered VS operating mode, we can write:

(24) $$R_{1}^{*}=R_{1.1}+R_{1.2}+R_{1.4}=\mathrm{const}\;\mathrm{and}\;R_{2}^{*}=R_{2.1}+R_{2.2}+R_{2.4}=\mathrm{const}.$$

In turn, based on (23) and (24), for the total magnetic resistances of the magnetic cores of MC_1_ and MC_2_, the following equations will be valid:

(25) $$R_{M1}=R_{1}^{*}+R_{1.3}\;\mathrm{and}\;R_{M2}=R_{2}^{*}+R_{2.3},$$

where $R_{1.3}$ and $R_{2.3}$ are, respectively, the magnetic resistances of the side sections of the magnetic cores of MC_1_ and MC_2_, the values of which vary under the disturbing impact *P*_α_(*t*).

The impedances of the electrical windings of MC_1_ and MC_2_ are determined as follows:

(26) $${\dot{Z}}_{1}=R_{1}+j\omega_{0}\times \frac{w_{1}^{2}}{{\dot{Z}}_{M1}}\;\mathrm{and}\;{\dot{Z}}_{2}=R_{2}+j\omega_{0}\times \frac{w_{2}^{2}}{{\dot{Z}}_{M2}},$$

where *R*_1_ and *R*_2_ are, respectively, the active resistances of the electrical windings of MC_1_ and MC_2_; ${\dot{Z}}_{M1}=R_{M1}+j\omega_{0}\times X_{M1}$ and ${\dot{Z}}_{M2}=R_{M2}+j\omega_{0}\times X_{M2}$ are, respectively, impedances of the electrical windings of MC_1_ and MC_2_; *X_M_*_1_ and *X_M_*_2_ are, respectively, parameters reflecting iron losses due to hysteresis and eddy currents.

Considering that iron losses are insignificant, i.e., $X_{M1}\langle \langle R_{M1}$ and $X_{M2}\langle \langle R_{M2}$ (${\dot{Z}}_{M1}=R_{M1}$ and ${\dot{Z}}_{M2}=R_{M2}$), Equation (26) can be rewritten as follows:

(27) $${\dot{Z}}_{1}=R_{1}+j\omega_{0}\times \frac{w_{1}^{2}}{R_{M1}}\;\mathrm{and}\;{\dot{Z}}_{2}=R_{2}+j\omega_{0}\times \frac{w_{2}^{2}}{{\dot{Z}}_{M2}},$$

Define the variable magnetic resistances of the side sections of the magnetic cores of MC_1_ and MC_2_:

(28) $$R_{1.3}=R_{1.3}^{\prime }+R_{1.3}^{\prime \prime }=\frac{l_{1.3}^{\prime }}{\mu^{*}\times S_{1.3}}+\frac{l_{1.3}^{\prime \prime }}{\mu_{0}\times (S_{1.3}+\Delta S)}=\frac{S_{1.3}\times (l_{1.3}^{\prime }\times \mu_{0}+l_{1.3}^{\prime \prime }\times \mu^{*})+l_{1.3}^{\prime }\times \mu_{0}\times \Delta S}{\mu^{*}\times S_{1.3}\times \mu_{0}\times (S_{1.3}+\Delta S)},\;R_{2.3}=R_{2.3}^{\prime }+R_{2.3}^{\prime \prime }=\frac{l_{2.3}^{\prime }}{\mu^{*}\times S_{2.3}}+\frac{l_{2.3}^{\prime \prime }}{\mu_{0}\times (S_{2.3}-\Delta S)}=\frac{S_{2.3}\times (l_{2.3}^{\prime }\times \mu_{0}+l_{2.3}^{\prime \prime }\times \mu^{*})-l_{2.3}^{\prime }\times \mu_{0}\times \Delta S}{\mu^{*}\times S_{2.3}\times \mu_{0}\times (S_{2.3}-\Delta S)},$$

where $R_{1.3}^{\prime }=\frac{l_{1.3}^{\prime }}{\mu^{*}\times S_{1.3}}$ and $R_{2.3}^{\prime }=\frac{l_{2.3}^{\prime }}{\mu^{*}\times S_{2.3}}$ are, respectively, the total magnetic resistances of the air gaps in the side sections of the magnetic cores of MC_1_ and MC_2_, $l_{1.3}^{\prime },\;S_{1.3}$ and $l_{2.3}^{\prime },\;S_{2.3}$ are, respectively, the length and average cross-sectional area of the side sections of the magnetic cores of MC_1_ and MC_2_, $\mu^{*}$ is the magnetic permeability of the material of the side sections of the magnetic cores of MC_1_ and MC_2_; $R_{1.3}^{\prime \prime }=\frac{l_{1.3}^{\prime \prime }}{\mu_{0}\times (S_{1.3}+\Delta S)}$ and $R_{2.3}^{\prime \prime }=\frac{l_{2.3}^{\prime \prime }}{\mu_{0}\times (S_{2.3}+\Delta S)}$ are the total magnetic resistances of the air gaps in the side sections of the magnetic cores of MC_1_ and MC_2_, $l_{1.3}^{\prime \prime },\;S_{1.3}$ and $l_{2.3}^{\prime \prime },\;S_{2.3}$ are, respectively, the length and average cross-sectional area of the air gaps in the side sections of the magnetic cores of MC_1_ and MC_2_, $\mu^{*}$ is the magnetic permeability of the material of the magnetic cores of MC_1_ and MC_2_, $\mu_{0}$ and ΔS are the magnetic permeability of the air and the variable portion of the cross-sectional area of the air gaps in the side sections of the magnetic cores of MC_1_ and MC_2_.

Given the aforementioned and according to (25), determine the total magnetic resistance of the MC_1_ magnetic core:

(29) $$\begin{array}{c} R_{M1}=R_{1}^{*}+\frac{S_{1.3}\times (l_{1.3}^{\prime }\times \mu_{0}+l_{1.3}^{\prime \prime }\times \mu^{*})+l_{1.3}^{\prime }\times \mu_{0}\times \Delta S}{\mu^{*}\times S_{1.3}\times \mu_{0}\times (S_{1.3}+\Delta S)}=\to \\ \to =\frac{R_{1}^{*}\times \mu^{*}\times S_{1.3}\times \mu_{0}\times S_{1.3}+S_{1.3}\times (l_{1.3}^{\prime }\times \mu_{0}+l_{1.3}^{\prime \prime }\times \mu^{*})+(R_{1}^{*}\times \mu^{*}\times S_{1.3}\times \mu_{0}+l_{1.3}^{\prime }\times \mu_{0})\times \Delta S}{\mu^{*}\times S_{1.3}\times \mu_{0}\times (S_{1.3}+\Delta S)} \end{array}$$

After simple transformations (29), we obtain:

(30) $$R_{M1}=\frac{K_{1}+l_{1.3}^{\prime }\times \mu_{0}+l_{1.3}^{\prime \prime }\times \mu^{*}+(K_{1}+l_{1.3}^{\prime }\times \mu_{0})\times \Delta S^{*}}{\mu^{*}\times \mu_{0}\times S_{1.3}\times \left(1+\Delta S^{*}\right)},$$

where $K_{1}=R_{1}^{*}\times \mu^{*}\times S_{1.3}\times \mu_{0}$ is the design factor of the MC_1_ magnetic core; $\Delta S^{*}=\frac{\Delta S}{S_{1.3}}$ is the relative change in the total air gap area under the disturbing impact *P*_α_(*t*).

The ratio for the total resistance of the MC_2_ magnetic circuit is determined similarly:

(31) $$R_{M2}=\frac{K_{2}+l_{2.3}^{\prime }\times \mu_{0}+l_{2.3}^{\prime \prime }\times \mu^{*}-(K_{2}-l_{2.3}^{\prime }\times \mu_{0})\times \Delta S^{*}}{\mu^{*}\times \mu_{0}\times S_{2.3}\times \left(1-\Delta S^{*}\right)},$$

where $K_{2}=R_{2}^{*}\times \mu^{*}\times S_{2.3}\times \mu_{0}$ is the design factor of the MC_2_ magnetic core; $\Delta S^{*}=\frac{\Delta S}{S_{2.3}}$ is the relative change in the total air gap area under the disturbing impact *P*_α_(*t*).

According to Equations (26) and (27), define the impedance of the MC_1_ electrical winding:

(32) $$\begin{array}{c} {\dot{Z}}_{1}=R_{1}+j\omega_{0}\times \frac{w_{1}^{2}\times \mu^{*}\times \mu_{0}\times S_{1.3}\times \left(1+\Delta S^{*}\right)}{K_{1}+l_{1.3}^{\prime }\times \mu_{0}+l_{1.3}^{\prime \prime }\times \mu^{*}+(K_{1}+l_{1.3}^{\prime }\times \mu_{0})\times \Delta S^{*}}=\to \\ \to =R_{1}+j\omega_{0}\times \frac{w_{1}^{2}\times \mu^{*}\times \mu_{0}\times S_{1.3}}{K_{1}+l_{1.3}^{\prime }\times \mu_{0}+l_{1.3}^{\prime \prime }\times \mu^{*}+(K_{1}+l_{1.3}^{\prime }\times \mu_{0})\times \Delta S^{*}}+\to \\ \to +j\omega_{0}\times \frac{w_{1}^{2}\times \mu^{*}\times \mu_{0}\times S_{1.3}\times \Delta S^{*}}{K_{1}+l_{1.3}^{\prime }\times \mu_{0}+l_{1.3}^{\prime \prime }\times \mu^{*}+(K_{1}+l_{1.3}^{\prime }\times \mu_{0})\times \Delta S^{*}}. \end{array}$$

Considering the relatively small values of the variable component, Equation (32) can be significantly simplified:

(33) $${\dot{Z}}_{1}=R_{1}+j\omega_{0}\times \frac{w_{1}^{2}\times \mu^{*}\times \mu_{0}\times S_{1.3}\times \Delta S^{*}}{K_{1}+l_{1.3}^{\prime }\times \mu_{0}+l_{1.3}^{\prime \prime }\times \mu^{*}},$$

The impedance of the MC_2_ electrical winding is determined similarly:

(34) $${\dot{Z}}_{2}=R_{2}-j\omega_{0}\times \frac{w_{2}^{2}\times \mu^{*}\times \mu_{0}\times S_{2.3}\times \Delta S^{*}}{K_{2}+l_{2.3}^{\prime }\times \mu_{0}+l_{2.3}^{\prime \prime }\times \mu^{*}},$$

Considering that *LC* converters operate in series resonance mode, we can write:

(35) $$\begin{array}{c} {\dot{U}}_{1}={\dot{I}}_{1}\times {\dot{Z}}_{1}=\frac{{\dot{U}}_{0}}{R_{1}}\times \left(R_{1}+j\omega_{0}\times \frac{w_{1}^{2}\times \mu^{*}\times \mu_{0}\times S_{1.3}\times \Delta S^{*}}{K_{1}+l_{1.3}^{\prime }\times \mu_{0}+l_{1.3}^{\prime \prime }\times \mu^{*}}\right)=\to \\ \to ={\dot{U}}_{0}+j\omega_{0}\times \frac{{\dot{U}}_{0}}{R_{1}}\times \frac{w_{1}^{2}\times \mu^{*}\times \mu_{0}\times S_{1.3}\times \Delta S^{*}}{K_{1}+l_{1.3}^{\prime }\times \mu_{0}+l_{1.3}^{\prime \prime }\times \mu^{*}};\\ {\dot{U}}_{2}={\dot{I}}_{2}\times {\dot{Z}}_{2}=\frac{{\dot{U}}_{0}}{R_{2}}\times \left(R_{2}-j\omega_{0}\times \frac{w_{2}^{2}\times \mu^{*}\times \mu_{0}\times S_{2.3}\times \Delta S^{*}}{K_{2}+l_{2.3}^{\prime }\times \mu_{0}+l_{2.3}^{\prime \prime }\times \mu^{*}}\right)=\to \\ \to ={\dot{U}}_{0}-j\omega_{0}\times \frac{{\dot{U}}_{0}}{R_{2}}\times \frac{w_{2}^{2}\times \mu^{*}\times \mu_{0}\times S_{2.3}\times \Delta S^{*}}{K_{2}+l_{2.3}^{\prime }\times \mu_{0}+l_{2.3}^{\prime \prime }\times \mu^{*}}. \end{array}$$

Based on the need for implementing the identity condition for the electrical windings and magnetic cores of MC_1_ and MC_2_, we can state the following:

*R* = *R*_1_ = *R*_2_; *w* = *w*_1_ = *w*_2_; *K* = *K*_1_ = *K*_2_; *S*_3_ = *S*_1.3_ = *S*_2.3_; $l_{3}^{\prime }=l_{1.3}^{\prime }=l_{2.3}^{\prime }$; $l_{0.3}^{\prime \prime }=l_{1.3}^{\prime \prime }=l_{2.3}^{\prime \prime }$.

Given the aforementioned, determine the differential data signal from the VS output:

(36) $$\Delta \dot{U}={\dot{U}}_{1}-{\dot{U}}_{2}=2\times j\omega_{0}\times \frac{{\dot{U}}_{0}}{R}\times \frac{w^{2}\times \mu^{*}\times \mu_{0}\times S_{3}\times \Delta S^{*}}{K+l_{3}^{\prime }\times \mu_{0}+l_{3}^{\prime \prime }\times \mu^{*}}=j\times \omega_{0}\times {\dot{U}}_{0}\times K_{C}\times \Delta S^{*}$$

where $K_{C}=\frac{2\times w^{2}\times \mu^{*}\times \mu_{0}\times S_{3}}{R\times (K+l_{3}^{\prime }\times \mu_{0}+l_{3}^{\prime \prime }\times \mu^{*})}$ is the generalized VS design factor.

From Equation (10), it follows that the imaginary component of the differential data signal gives the information on the VS’ response to the external disturbance *P*_α_(*t*):

(37) Im(ΔU˙)=ω0×U˙0×KC×ΔS∗.

Using the synchronous detection method, at the quadrature synchronous detector output, we obtain:

(38) $$\Delta U_{\mathrm{II}D}(t)=K_{\mathrm{II}D}\times \left|\Delta \dot{U}\right|\times \sin(\arg\Delta \dot{U})=K_{\mathrm{II}D}\times K_{C}\times \omega_{0}\times U_{0}\times \Delta S^{*}(t),$$

where *K_D_* is the quadrature synchronous detector conversion factor.

Define the functional dependence of the variable component of the air gap area on the inertial element rotation angle θ:

(39) $$\Delta S^{*}=l_{S}\times h_{S}=\frac{\pi \times r\times h_{S}\times \theta }{180}=K_{S}\times \theta ,$$

where $l_{S}=\frac{\pi \times r\times \theta }{180}$ and *h_S_* are, respectively, the tooth rim profile arc length and pole tip height; $K_{S}=\frac{\pi \times r\times h_{S}}{180}$ is the tooth rim design factor; *r* is the tooth rim radius;

Eventually, for the case under consideration, we obtain:

(40) $$\Delta U_{\mathrm{II}D}(t)=K_{\mathrm{II}D}\times K_{C}\times K_{S}\times \omega_{0}\times U_{0}\times \theta (t),$$

According to Equation (40), determine the rotation angle of the annular sensing element in the form of an internal tooth rim relative to the VS housing:

(41) $$\theta (t)=\Delta U_{\mathrm{II}D}(t)\times {\left(K_{\mathrm{II}D}\times K_{C}\times K_{S}\times \omega_{0}\times U_{0}\right)}^{-1},$$

Equation (41) determines the θ(*t*) change pattern, according to which, using the solution to the differential Equation (21), the acceleration $\frac{d^{2}\alpha }{dt^{2}}$ of the VS housing displacement in the absolute coordinate system is ultimately found.

In general, according to (21), the disturbing impact *P*_α_(*t*) on an object causes a complex oscillatory nature of its linear displacement:

(42) $$\alpha (t)=\sum_{i=1}^{n}\alpha_{i}\times \sin(\Omega_{i}t+\varphi_{i}).$$

It should be noted that the tooth rim inertial mass together with the elastic suspension also form a high-Q resonant mechanical system. Therefore, at the disturbing impact *P*_α_(*t*), the tooth rim oscillation will be excited at the resonant frequency $\Omega_{\mathrm{II}}$ of this mechanical system. In this case, at a sudden disturbing impact *P*_λ_(*t*) on an object, free torsional tooth rim oscillations around the VS’ vertical axis of symmetry arise, causing corresponding angular deviations, which can be conventionally represented as follows:

(43) $$\theta (t)=\theta_{\max}\times \sin(\Omega_{\mathrm{II}}t+\varphi_{\theta }),$$

where θ*_max_* is the maximum angular tooth rim displacement during torsional oscillations relative to its neutral position; Ω_II_ is the resonant angular core displacement frequency; φ_δ_ is the AP’s initial displacement phase.

In this case, for the recorded data signal, we can write:

(44) $$\Delta U_{\mathrm{II}D}(t)=K_{\mathrm{II}D}\times K_{C}\times K_{S}\times \omega_{0}\times U_{0}\times \times Im\left[{\dot{\theta }}_{\max}\times e^{j\Omega_{\mathrm{II}}t}\right].$$

The resulting Equation (44) reflects a functional dependence of the increment of the measuring bridge output voltage imaginary component on the AP linear displacement caused by external vibrations, the parameters of the temporary implementation of which indirectly reflect the processes occurring in the environment.

## 6. Analyzing the VS Operation in the Deviation Angle ψ Recording (Inclinometer) Mode

Consider the specifics of the VS operation in the mode of recording a possible angular deviation ψ of the VS’ vertical axis of symmetry from the vertical axis Z of the absolute coordinate system, which coincides with the vertical component *H*_0_ of the background geomagnetic field, oriented, in turn, in the gravity direction. When analyzing the specifics of this VS operating mode, use elements of the parametric theory of fluxgates and assume that the main magnetic flux, generated by the corresponding excitation current of each measuring coil, is concentrated in the central sections of their magnetic cores.

In fact, this is the fluxgate operating mode, in which the magnetic permeability parameter μ* of the ferrite bushing AP, where an alternating magnetic field *H*(*t*) = *H*_~_ with a constant amplitude affects the VS’ sensitive element. In other words, this mode implements the modulation of the AP magnetic permeability μ* by an alternating magnetic field H_~_, the result of which can be written as follows:

μ* = μ*(*H*_~_) = μ*(*t*). (45)

In addition, under natural conditions, the VS’ sensitive element is also affected by the vertical component *H*_0_ of the background geomagnetic field, the current value of which is read by the VS in a fluxgate mode. In this case, any change in the angular deviation ψ of the VS’ vertical axis of symmetry from the vertical axis *Z* of the absolute coordinate system will correspondingly change the recorded value of the vertical component *H*_0_ of the background geomagnetic field, i.e., the magnitude of the projection of this field onto the VS’ vertical axis of symmetry will be measured.

**Figure 9:**
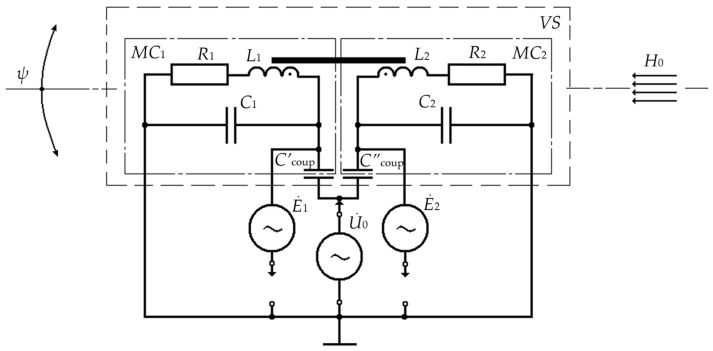
Equivalent Circuit of the VS in an Inclinometer Mode.

Figure 9 shows the circuit wiring diagram of the VS operating in the fluxgate mode.

When identical electrical windings of MC_1_ and MC_2_ are powered with an exciting sine-wave voltage *u*_0_(*t*) = *U*_0_ × sinω_0_*t*, corresponding electric currents ${\dot{I}}_{1}$ and ${\dot{I}}_{2}$ arise in them, inducing magnetizing forces *F*_1_ = *I*_1_ × *w*_1_ and *F*_2_ = *I*_2_ × *w*_2_ inside the AP’s ferrite sleeve, which is a common element of the central sections of the magnetic cores of MC_1_ and MC_2_.

In turn, the magnetizing forces *F*_1_ = *I*_1_ × *w*_1_ and *F*_2_ = *I*_2_ × *w*_2_ induce corresponding magnetic fields with corresponding magnetic induction vectors inside the AP’s ferrite sleeve:

*B*_1_ = *f*(*H*_0_ + *H*_~_) and *B*_2_ = *f*(*H*_0_ − *H*_~_), (46)

where *H*_~_ = *H_m_* × sinω_0_*t* is the strength of the auxiliary alternating (excitation) field induced by each of the magnetizing forces *F*_1_ and *F*_2_ inside the AP’s ferrite sleeve; *H*_0_ is the strength of the vertical component of the background constant geomagnetic field.

The total vector of magnetic induction inside the AP’s ferrite sleeve:

*B*_Σ_ = *B*_1_ + *B*_2_. (47)

Conduct a third-order polynomial approximation of the *B* = *f*(*H*_∑_) dependence:

*B* = *aH*_∑_ − *bH*^3^_∑_. (48)

where *a* and *b* are positive approximation coefficients depending on the core material and shape; *H*_∑_= *H*_0_ ± *H*_~_;

Considering that MC_1_ and MC_2_ are in a uniform background constant geomagnetic field and according to (46) and (47), we can write:

*B*_1_ = *a* × *H*_0_ + *a* × *H*_~_ − *b* × (*H*_0_)^3^ − 3 × *b* × (*H*_0_)^2^ × *H*_~_ − 3 × *b* × *H*_0_ × (*H*_~_)^2^ − *b* × (*H*_~_)^3^; *B*_2_ = *a* × *H*_0_ − *a* × *H*_~_ − *b* × (*H*_0_)^3^ + 3 × *b* × (*H*_0_)^2^ × *H*_~_ − 3 × *b* × *H*_0_ × (H_~_)^2^ + *b* × (H_~_)^3^. (49)

Then, according to (47) and (49), we ultimately obtain:

*B*_1_ + *B*_2_= 2 × *a* × *H*_0_ − 2 × *b* × (*H*_0_)^3^ − 6 × *b* × *H*_0_ × (*H*_~_)^2^. (50)

The last term in (50) contains the product of the strengths of the constant external background *H*_0_ and the auxiliary alternating *H*_~_ magnetic fields and is responsible for the generation of magnetic modulation EMFs in the electrical windings of MC_1_ and MC_2_, which can be represented as follows relative to the grounded ends of these windings:

*e*_1_(*t*)|*_H_*_o=const≠0_ = 6 × *b* × *s* × *w*_1_ × *H*_0_ × [d(*H*_~_)^2^/d*t*] ≠ 0; *e*_2_(*t*)|*_H_*_o=const≠0_ = −6 × *b* × *s* × *w*_2_ × *H*_0_ × [d(*H*_~_)^2^/d*t*] ≠ 0. (51)

Considering that the auxiliary variable field *H*_~_ = *H*_m_ × sinω*t*, Equation (51) for the VS case with mutually parallel *H*_~_ and *H*_0_ fields (at *H*_0_ = const ≠ 0 and *H*_m_ >> *H*_0_) can be transformed as follows:

*e*_1_(*t*) = 6 × *b* × *s* × ω_0_ × *w*_1_ × *H*_0_ × *H*^2^_m_ × sin2ω_0_t and *e*_2_(*t*) = −6 × *b* × *s* × ω_0_ × *w*_2_ × *H*_0_ × *H*^2^_m_ × sin2ω_0_*t* (52)

Equation (52) shows that the output EMF has a frequency multiple of that of the auxiliary alternating field ω_0_.

Thus, we can state that in the presence of a magnetizing field with the magnitude equal to the projection *H*_0_ of the external background geomagnetic field onto the longitudinal axis of the AP ferrite sleeve (along the vertical coordinate axis *Z*), double frequency EMFs are induced in the electric windings of MC_1_ and MC_2_, the phases of which shift by 180^0^ when the external field changes oppositely, and the magnitude of these double frequency EMFs varies over a wide range in direct proportion to the strength component *H*_0_ of the external magnetic field.

When the VS operates in the fluxgate mode, data signals are also read from the output terminals of the lower row of the electrical windings of MC_1_ and MC_2_ as corresponding induction EMFs *e*_1_(*t*) and *e*_2_(*t*), and the measured value is the difference:

Δ*U*_Σ_(*t*) = *e*_1_(*t*) + *u*_1_(*t*) − [−*e*_2_(*t*) + *u*_2_(*t*)] = 12 × *b* × *s* × *ω*_0_ × *w* × *H*_0_ × *H_m_*^2^ × sin2*ω_0_t*, (53)

where *w*_1_ = *w*_2_ = *w*; *u*_1_(*t*) = *u*_2_(*t*) are electrical output signals of corresponding *LC* converters powered by the sine-wave signal generator voltage *u*_0_(*t*).

When the VS’ longitudinal axis deviates from the vertical axis *Z* of the absolute coordinate system by an angle ψ, the corresponding component *H*_0_ starts acting on the VS’ sensitive element P in the form of *H*_0_ × cosψ × sin2ω_0_*t.*

Then, given the aforementioned, we can write:

Δ*U*_Σ_(*t*) = 12 × *b* × *s* × ω_0_ × *w* × *H*_0_ × *H*^2^_m_ × cosψ × sin2ω_0_*t*. (54)

After implementing the corresponding synchronous detection procedure with a commutation vector at double frequency ω_0_, the quadrature detector output data signal can be represented as follows:

Δ*U*_III*D*_(*t*) = 12 × *K*_III*D*_ × *b* × *s* × ω_0_ × *w* × *H*_0_ × *H*^2^_m_ × cosψ, (55)

where *K*_III*D*_ is the quadrature detector conversion factor.

Analysis of Equation (53) confirms the possibility of using the considered VS version in an inclinometer mode.

Generalizing the results for the VS operation simultaneously in all three modes, we obtain the following equation for the total output data signal:

(56) ΔUΣ(t)=ΔUID(t)+ΔUIID(t)+ΔUIIID(t)=−ω0×KID×Q×⋅U0×Imδ˙max×ejΩIt+→→+KIID×KC×KS×ω0×U0×Imθ˙max×ejΩIIt+12×KIID×b×s×ω0×w×H0×Hm2×cosψ.

Selective filtering with three filters tuned to frequencies Ω_I_, Ω_II_, and 2ω_0_ allows for obtaining, respectively, the required current δ, θ, and ψ values.

## 7. Experimental Studies of the Vibration Sensor

Experimental studies have been planned and performed to prove the viability of the idea of building a vibration sensor with expanded functionality based on the new design solution. All implemented physical experiments were aimed at identifying the fundamental possibility of practically implementing three combined operating modes in a single vibration sensor. The experiment identified and studied physical effects, manifested as a response of the vibration sensor’s sensitive elements to various vibrational disturbances and changes in its inclination angle. Since we tested a prototype vibration sensor model, developed based on new design principles, the experimental study was not supposed to determine any precision parameters of that sensor.

The vibration sensor will be further optimized from the process, design, and schematic standpoint with subsequent corresponding metrological assessment of its technical parameters.

**Figure 10:**
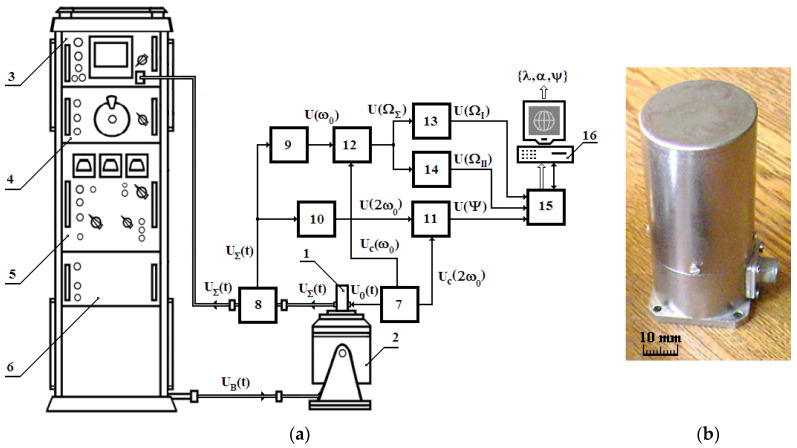
Experimental Bench: (**a**)—generalized structural block diagram; (**b**)—general view of the vibration sensor.

To study the dynamic properties of the vibration sensor, a special experimental bench was used, comprising a vibration sensor 1, an electrodynamic-type vibration unit (EVU), and the data signal electronic processing module (EPM) (Figure 10a,b).

The EVU comprises an electrodynamic shaker 2 and a control rack SUPV-0.1, generating the electrical signal U_B_(t) to control the shaker 2 operating modes and consisting of the following functional modules: 3—acceleration measurement module; 4—sine-wave generator module; 5—amplifier module; 6—magnetization module. In turn, the electronic signal processing module comprises the following functional elements: 7—vibration sensor excitation sine-wave voltage *U*_0_(*t*) generator with the cyclical frequency ω_0_ and synchronization voltages *U_C_*(ω_0_) and *U_C_*(2ω_0_); 8—splitter; 9 and 10—input selective amplifiers; 11 and 12—quadrature detectors; 13 and 14—output selective amplifiers-detectors; 15—data collection system (DCS) E502 by LCard; 16—PC.

To determine the dynamic characteristics of the vibration sensor based on digital spectral analysis, a series of experiments were planned and performed on this bench. The experimental studies considered that for multiple exciting forces with different frequencies, acting simultaneously in the system, the resulting vibration will be the sum of vibrations at each frequency. In this case, the resulting temporal oscillations of vibration sensor 1 will have a complex change pattern.

All experiments were performed at the vibration sensor’s excitation signal amplitude voltage of *U*_0_ = 10 V and a frequency of *f*_0_ = ω_0_/2π = 6 kHz.

Spatial displacements of vibration sensor 1 were excited by the electrodynamic shaker 2, which generated both vertical linear and horizontal torsional oscillations of vibration sensor 1, controlled by the electrical signal *U_B_*(t) from the control rack. In this case, the complex-shaped data signal *U*_Σ_(t) was read at the vibration sensor 1 output, which was then fed through splitter 8 to the inputs of module 3 and selective amplifiers 9 and 10. The extracted electric signals *U*(ω_0_) and *U*(2ω_0_) from, respectively, the outputs of selective amplifiers 9 and 10, were fed to the inputs of, respectively, quadrature detectors 12 and 11, whose synchronization inputs received synchronization voltages *U_C_*(ω_0_) and *U_C_*(2ω_0_) from generator 7. The complex-shaped electric signal *U*(Ω_Σ_) from the quadrature detector 12 output was fed to the inputs of output selective amplifiers-detectors 13 and 14, and then, after appropriate conversion, as electric data signals *U*(Ω_I_) and *U*(Ω_II_) to the corresponding inputs of DCS 15. Thereat, the electric signal *U*(Ψ) from the quadrature detector 11 output was also fed to the corresponding input of DCS 15.

It should be noted that DCS 16 performs analog-to-digital conversion of three data signals *U*(Ψ), *U*(Ω_I_), and *U*(Ω_II_), providing information on the angular inclination Ψ of vibration sensor 1 relative to the horizontal component of the geomagnetic field, linear displacement λ of the rod-shaped sensitive element of vibration sensor 1, and angular displacement *θ* at rotational oscillations of the tooth rim sensitive element of that sensor. Furthermore, DCS 16, functionally interacting with module 16, performs digital filtration and normalization of data signals. In turn, module 16, implementing the corresponding algorithms for processing the incoming data array, determines the specific values of parameters λ (linear displacement of the vibration sensor), α (angular displacement at rotational oscillations of the vibration sensor), and Ψ (the vibration sensor inclination angle).

**Figure 11:**
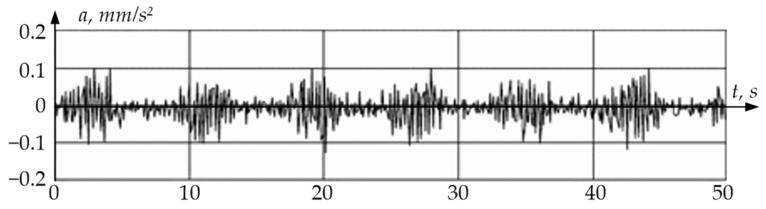
Data Signal Oscillogram.

The measurement result was a set of time-digital sequences of vibration accelerations *a* = *f*[*n*] (see Figure 11). Discrete values of vibration accelerations were recorded at the *t*_0_, *t*_1_, *t*_2_, …, *t*_N−1_ time instants.

A sampling period τ = *T*/*N* = 2.5 ms was adopted, where *T* = 50 ms is the main oscillation period, and *f*_τ_ =1/τ= 400 Hz is the cutoff frequency (Nyquist frequency [32]). In this case, the maximum expected frequency should be twice less than *f*_τ_, i.e., *f_max_* = 200 Hz.

The dependence *a* = *f*[*n*] = *f*(*n* × τ) was integrated twice. Herewith, the resulting amplitude-time-digital sequence *w*[*n*] underwent numerical Fourier transforms using the MathCAD 2000 software package:

(57) $$W[f]=\tau \times \sum_{n=0}^{N-1}w[n]\times \exp\left(-i\times 2\pi \times f\times n/N\right),$$

where *w*[*n*] = *w*(*n* × τ), *n* = 0, 1, …, *N* − 1; $i=\sqrt{-1}$.

Then the power spectral density (PSD) was determined:

(58) $$P(f)=\frac{\tau }{N}\times {\left|\sum_{n=0}^{N-1}w[n]\times \exp\left(-i\times 2\pi \times f\times n/N\right)\right|}^{2}.$$

It is known that the sample spectrum gives statistically inconsistent PSD estimates. As a rule, there are false peaks, the position of which changes depending on the time origin coordinate. In this regard, one of the most effective smoothing methods, the Welch method, was used.

The *w*[*n*] data was divided into *K* segments, D samples each, with an *S* shift between adjacent segments (*S* ≤ *D*). The maximum number of segments *K* was determined by the integer part of the number *K* = (*N* − *D*)/(*S* + 1). Each segment was ‘weighted’ using the Nuttall window. Averaging over periodograms of segments gave the final PSD estimate:

(59) $$P(f)=\frac{1}{K}\times \sum_{k=0}^{K-1}P_{k}(f).$$

A logarithmic scale was used to present the results graphically. The difference in PSD levels in dB was plotted along the ordinate:

(60) $$\tilde{P}(f)=10\times \mathrm{lg}\left[\frac{P(f)}{\max\left\{P(f)\right\}}\right].$$

**Figure 12:**
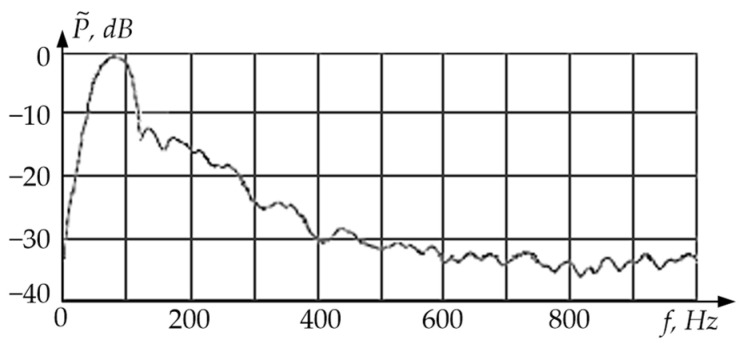
Spectral Analysis of the Vibration Sensor Signal at Linear Oscillations.

Figure 12 shows the digital spectral analysis results for a vibration sensor with excitation of a single rod-shaped sensitive element on flat membrane elastic suspensions (linear oscillations with a frequency *f*_I_ = Ω_I_/2π ≈ 100 Hz).

**Figure 13:**
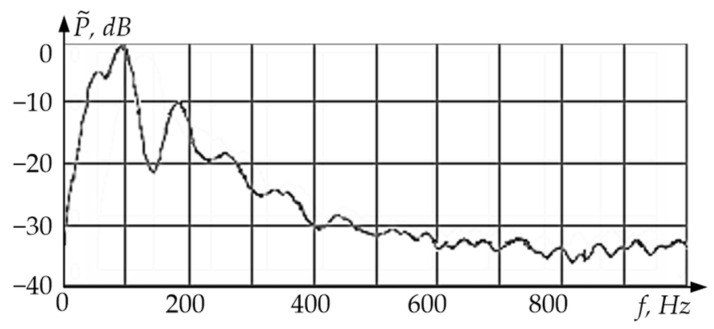
Spectral Analysis of the Vibration Sensor at Combined Oscillations.

Figure 13 shows the digital spectral analysis results for a vibration sensor with simultaneous excitation of two resonant elements, i.e., a rod on flat membrane elastic suspensions (linear oscillations with a frequency *f*_I_ = Ω_I_/2π ≈ 100 Hz) and a tooth rim on flat beam elastic suspensions (torsional oscillations with a frequency *f*_II_ = Ω_II_/2π ≈ 200 Hz).

Figure 13 shows the spectral components of linear and torsional oscillations, which correspond to the resonant cyclic frequencies Ω_I_ and Ω_II_ of corresponding sensitive elements on elastic suspensions. This fact indicates the possibility of simultaneous recording of different vibration types with a single vibration sensor.

**Figure 14:**
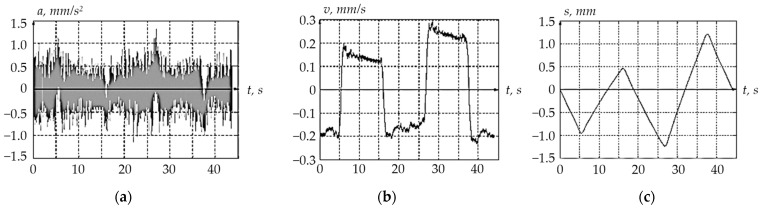
Fragments of the Excited Vibration Sensor Output Electrical Signals: (**a**) is a fragment of the original electrical signal from acceleration; (**b**) is a fragment of the electrical speed signal obtained during the first integration of the acceleration signal; (**c**) is a fragment of the electrical displacement signal obtained by reintegrating the acceleration.

When vibration sensor 1 was excited using shaker 2, the corresponding electrical signals were read at its output, the nature of which is shown in Figure 14.

**Figure 15:**
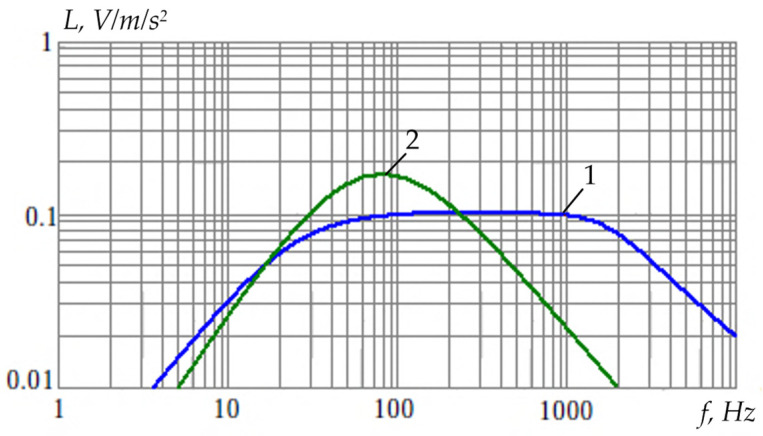
The Vibration Sensor AFR in Various Excitation Modes.

Also, in the vibration sensor experimental studies, its amplitude-frequency response (AFR) was obtained for two operating modes (see Figure 15): 1—mode of recording torsional oscillations of the tooth rim sensitive element relative to the suspension point; 2—mode of recording linear oscillations of the rod-shaped sensitive element.

During the experiment, the vibration sensor response to changes in its inclination angle ψ relative to the Earth’s gravitational field or the vertical component of its magnetic field was also studied. For this purpose, a special rotating device of electrodynamic shaker 2 was used, on which an indicator of a theodolite limb was installed with a vernier scale allowing to set any inclination angle with an accuracy of 0.1 degrees of arc.

**Figure 16:**
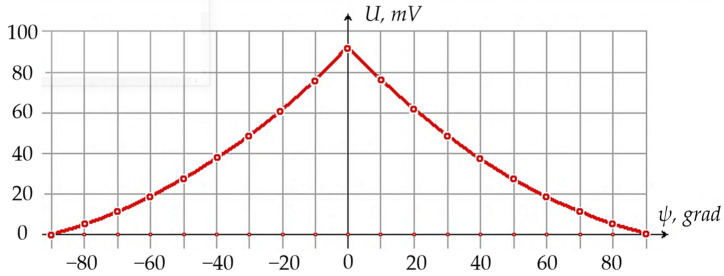
Calibration Curve of the Vibration Sensor in the Inclinometer Mode.

In this case, when varying the vibration sensor inclination angle ψ, corresponding changes in its original output signal were observed. Figure 16 shows the results of this part of the experiment.

Note that the differential connection of measuring coils and the features of the vibration sensor excitation mode facilitate the invariant measurement of controlled parameters relative to the ambient temperature and humidity.

Also note that the design and functional features of the hybrid vibration sensor do not affect the level of integration compatibility with other systems and monitoring devices. The lack of excitation coils and permanent magnetic typical for conventional vibration sensors minimizes any active effects on the environment and various nearby equipment.

Thus, the experiments conducted with the vibration sensor model confirmed the operability and effectiveness of the proposed combination of controlling various physical parameters of vibration and measuring the spatial positioning angle of structure and objects using one hybrid sensor. At this research stage, the goal was to justify the new operating principle for the vibration sensor used in the original technical solutions proposed by the authors. Note that technical and economic problems were not tackled at this stage of work. They will be addressed in further research papers.

## 8. Conclusions

The analysis of the hybrid vibration sensor experimental study results confirmed the possibility of using it for recording various types of vibration disturbances and changes in the inclination angle of process equipment, arising during its operation.

The proposed version of the new type of hybrid vibration sensor is, in fact, a universal vibration sensor with expanded functionality, capable of simultaneously recording parameters of linear and torsional oscillatory displacements of the monitored object and controlling its inclination angle relative to the Earth’s gravitational field or the vertical component of its magnetic field. Basically, the developed vibration sensor combined the functions of three types of measuring transducers: 1—the linear vibration transducer (to register the linear movements of the vibrating object); 2—the pendulum vibration transducer (to register the rotation angle of the vibrating object during torsional oscillations); 3—the inclinometer (to control the absolute or relative angular position of the vibration object relative to the vertical). One such multi-purpose vibration sensor can replace three specialized sensors, which simplifies the installation of the sensor on the controlled object, the performance and processing of control operations, as well as the entire diagnostic system structure, its installation, and setup. All of the above improves the efficiency of control operations. Note that this paper does not provide an ultimate solution to all problems associated with the development and industrial usage of the suggested hybrid sensors because it only lays the theoretical foundation for the subsequent production of hybrid sensor prototypes, and their testing and comparison with the existing sensors based on technical and economic parameters.

The proposed vibration sensor version may further replace a whole range of modern vibration sensors for various purposes. The existing potential of this vibration sensor also allows for its wide implementation in measuring azimuth and inclination angle at the drilling well displacement in mining; monitoring the safe operation of specialized large vehicles and earthmoving machinery; monitoring the condition of tunnels, bridges, pipelines, as well as platforms, foundations, supports, trusses, girders, and antenna mast structures in construction and architecture.

To sum it up, we can state that along with the control over the object’s deviation from the vertical in monitoring systems to stabilize its angular position, the NDT issues will also be addressed, which will significantly improve the reliability of process objects and reduce the rate of possible emergencies in general.

## Data Availability

Data is contained within the article.
